# Propolis Is an Efficient Fungicide and Inhibitor of Biofilm Production by Vaginal *Candida albicans*

**DOI:** 10.1155/2015/287693

**Published:** 2015-03-01

**Authors:** Isis Regina Grenier Capoci, Patrícia de Souza Bonfim-Mendonça, Glaucia Sayuri Arita, Raphaela Regina de Araújo Pereira, Marcia Edilaine Lopes Consolaro, Marcos Luciano Bruschi, Melyssa Negri, Terezinha Inez Estivalet Svidzinski

**Affiliations:** ^1^Division of Medical Mycology, Teaching and Research Laboratory in Clinical Analyses, Department of Clinical Analysis of State University of Maringá, Avenida Colombo 5790, 87020-900 Maringá, PR, Brazil; ^2^Laboratory of Research and Development of Drug Delivery Systems, Department of Pharmacy, State University of Maringá, Maringá, PR, Brazil; ^3^Clinical Cytology Laboratory, Department of Clinical Analysis and Biomedicine, State University of Maringá, PR, Brazil

## Abstract

Vulvovaginal candidiasis (VVC) is one of the most common genital infections in women. The therapeutic arsenal remains restricted, and some alternatives to VVC treatment are being studied. The present study evaluated the influence of a propolis extractive solution (PES) on biofilm production by *Candida albicans* isolated from patients with VVC. Susceptibility testing was used to verify the minimum inhibitory concentration (MIC) of PES, with fluconazole and nystatin as controls. The biofilm formation of 29 vaginal isolates of *C. albicans* and a reference strain that were exposed to PES was evaluated using crystal violet staining. Colony-forming units were evaluated, proteins and carbohydrates of the matrix biofilm were quantified, and scanning electron microscopy was performed. The MIC of PES ranged from 68.35 to 546.87 *μ*g/mL of total phenol content in gallic acid. A concentration of 546.87 *μ*g/mL was able to cause the death of 75.8% of the isolates. PES inhibited biofilm formation by *C. albicans* from VVC. Besides antifungal activity, PES appears to present important antibiofilm activity on abiotic surfaces, indicating that it may have an additional beneficial effect in the treatment of VVC.

## 1. Introduction

Vulvovaginal candidiasis (VVC) is a frequently reported gynecological condition during the lives of healthy women. The literature shows that approximately 5–8% of women will develop a recurrent form of VVC [1], which has a significant effect on quality of life and poses a substantial burden to the healthcare system. Treatment and management costs for VVC are reported to be approximately USD$1 billion per year in the United States. Uncomplicated VVC cases are defined as single episodes that usually respond to treatment, whereas idiopathic recurrent VVC (RVVC) episodes are commonly untreatable [2].

The therapeutic arsenal that is available for VVC treatment is limited, and fluconazole and nystatin are the most frequently used [3]. However, nystatin has little therapeutic effect, and fluconazole is associated with the development of resistance by non-*Candida albicans Candida* species [4]. Amphotericin B may be an excellent therapeutic resource because of its high efficacy, but it has very high toxicity [5].

Alternatives to the use of commercial antifungal agents are natural products. Several factors have contributed to the development of medical practices that include medicinal plants, especially those that are inexpensive and easily handled [6].

Natural products, such as propolis, have been described as a promising option. It is a resin containing a complex mixture of substances, produced by honey bees, that results from the collection of substances secreted by plants, with the purpose of sealing and protecting the hive. Its chemical composition is complex, varying ecological characteristics of the region where it is collected [7]. Despite its complexity and variation, the biological activities are already well defined in the propolis studied worldwide, which have their standardized extracts with chemical composition determined [8]. Therapeutic properties, such as antimicrobial, anti-inflammatory, immunostimulatory, healing, and antiseptic effects, have been described in the literature [7–9].

In addition to factors related to therapy, several virulence factors appear to be responsible for VVC. Among these is the ability to adhere to human cells. Biofilm formation is closely related to the prolonged stay of these microorganisms in the vaginal cavity and is predictive of infection [10]. Evaluating the ability of biofilm formation is important in VVC because of the possibility of its occurrence in intrauterine devices (IUDs) and contraceptive vaginal rings [11, 12]. Moreover, biofilm is important in VVC and RVVC that are not related to the IUD because some fungal cells may remain in the vaginal mucosa together with a variety of other microorganisms that are organized in the form of biofilm. These yeasts have greater resistance to conventional antifungal therapy and may be responsible for the noneradication of* Candida* in the vaginal lumen, thus at least partially explaining the occurrence of RVVC [11].

Thus, knowing the problems associated with VVC, the present study evaluated the antifungal activity of propolis against* C. albicans* obtained from VVC and its potential to inhibit biofilm formation as a possible preventive therapeutic strategy for the treatment of VVC.

## 2. Materials and Methods

### 2.1. Propolis

Propolis was collected from hives of* Apis mellifera* L. bees at the apiary located in Cianorte (Parana, Brazil). The samples were frozen, tritured,and stored at −20°C until use [13].

### 2.2. Preparation of Propolis Extractive Solution

Propolis extractive solution (PES) was prepared by turbo extraction (3500 rpm) three times for 15 min at intervals of 5 min, with a propolis/ethanol ratio of 30/70 (w/w). The PES was filtered through filter paper, and the final initial weight was adjusted with ethanol [14].

### 2.3. Determination of Total Phenol Content

The total phenol content (TPC) of PES was determined by the Folin-Ciocalteu method [15] with some modifications [16]. The PES (2.0 *μ*L) was mixed with 1.0 mL Folin-Ciocalteu and 10.0 mL of water, with the final volume of 25 mL adjusted with 14.06% Na_2_CO_3_ (w/v). After 15 min, absorbance was read with a Shimadzu UV-1650PC spectrophotometer (Tokyo, Japan) at a wavelength of 760 nm. A calibration curve with solutions of gallic acid was used as a reference. The TPC is expressed as a percentage of total phenolic substances in PES and corresponds to the mean of six replicates.

### 2.4. *Candida albicans* Isolates and Growth Conditions

To test susceptibility and determine the total biomass of the biofilms on abiotic surfaces, we used the ATCC90028 reference strain of* C. albicans* from the American Type Culture Collection and 29* C. albicans* isolates from patients with VVC that belong to the archive collection of the Laboratory of Medical Mycology,* Universidade Estadual de Maringá*, Brazil.

In each experiment, the isolates were subcultured on Sabouraud Dextrose Agar (SDA; Difco) overnight at 37°C and then in CHROMágar* Candida*. The cellular density was adjusted using a Neubauer chamber before each assay.

### 2.5. Antifungal Assays

For susceptibility testing, we used the broth microdilution method according to the standards of the Clinical and Laboratory Standards Institute (M27-A3) [17], with some modifications for natural products [18]. We used RPMI 1640 (Roswell Park Memorial Institute, Gibco) with L-glutamine (without sodium bicarbonate) and 0.165 M 3-N morfolinopropanosulfônico (pH 7.2) as the buffer (Sigma), supplemented with 2% glucose. The final cellular density of the yeast was adjusted to 2.5–5 × 10^3^ colony-forming units (CFU)/mL in RPMI. The test was performed in flat-bottom 96-well microtiter plates (Techno Plastic Products, Switzerland). For the assay with PES, we tested concentrations of 34.17, 68.35, 136.71, 273.43, 546.87, 1093.75, 2187.5, 4375, 8750, and 17500 *μ*g/mL of total phenol content expressed in gallic acid. The plates were incubated at 35°C with shaking (70–80 rpm) for 48 h. Readings were performed with a visual reflection mirror. The minimum inhibitory concentration (MIC) of PES was considered the lowest concentration at which no fungal growth was evident. The minimum fungicidal concentration (MFC) was also determined by inoculating each concentration from the MIC test into plates that contained SDA. The plates were then incubated at 35°C for 24 h. The MFC was defined as the lowest concentration of PES that prevented yeast growth. For the antibiofilm assays, we used a subinhibitory concentration (0.5 × MIC) of PES (preconcentration MIC). This concentration was previously shown to be able to change the phenotypic and genotypic characteristics of the yeast, without affecting viability [19].

The antifungals fluconazole (Pfizer, Brazil) and nystatin (Sigma, St. Louis, MO, USA) were used. Serial dilutions were made with diluents that were appropriate in accordance with the M27-A3 guidelines of the CLSI, and microdilution testing was performed in accordance with the same document. Readings were performed on a microplate reader (Expert Plus, ASYS, UK) at 450 nm after 48 h of incubation. The MIC was defined as the lowest concentration of the antifungal agent that was able to inhibit 50% fluconazole and 90% nystatin relative to the positive control without drugs. As defined by the CLSI, negative controls (medium only), positive controls (medium and yeast), and the reference strain (*C. albicans* ATCC90028) were used in each test.

The cut-off levels of susceptibility to fluconazole and nystatin were utilized according to CLSI supplement M27-S3 [20] and Dalben-Dota et al. [18] to identify strains as susceptible (*S*), dose-dependent susceptible (DDS), and resistant (*R*): fluconazole (*S* ≤ 8 *μ*g/mL, DDS = 16–32 *μ*g/mL, *R* ≥ 64 *μ*g/mL), nystatin (*S* ≤ 4 *μ*g/mL, DDS = 8–32 *μ*g/mL, *R* ≥ 64 *μ*g/mL).

### 2.6. Cytotoxicity Assay

For the cytotoxicity experiments, HeLa cells (cervix adenocarcinoma cell line) donated by Dr. Luísa Lina Villa, ICESP-USP, São Paulo, Brazil, were cultured at 37°C under 5% CO_2_ in Dulbecco's modified Eagle's medium (DMEM; Gibco) that contained 10% fetal bovine serum (Gibco) and 1% penicillin/streptomycin (P/S; Gibco). After achieving 80% confluence, the cells were detached using 25% trypsin-ethylenediamine tetra-acetic acid (EDTA) solution (Gibco). The cell concentration was adjusted to 2 × 10^5^ cell/mL with fresh DMEM without P/S, and the suspension was added to the wells of a 96-well plate. Prior to the cytotoxicity assays, the wells were washed twice with phosphate-buffered saline (PBS), and PES at a MIC concentration was added to the cells and incubated overnight at 37°C under 5% CO_2_. Cells that were treated with the corresponding percentage of ethanol were used as a control. Afterward, cytotoxicity with PES was assessed using the Cell Titer 96 assay (Promega, Madison, WI, USA), based on the reduction of MTS (3-[4,5-dimethylthiazol-2-yl]-5-[3-carboxymethoxyphenyl]-2-[4-sulfophenyl]-2H-tetrazolium) in DMEM without phenol red. MTS is bioreduced by human epithelial cells into a formazan product that is soluble in tissue culture medium. After 3 h incubation at 37°C in the dark, the absorbance of formazan was measured at 490 nm using ASYS (Biochrom, Holliston, MA, USA). A control was performed by measuring the cellular activity of human cells grown under the same conditions but in the absence of PES. The cytotoxicity of the compound is presented as the average of three independent experiments with three replicates [21]. The percentage of cell viability (%CV) was calculated by the following equation: %CV = (*A*_sample_/*A*_blank_) × 100, where blank is the medium with cells and MTS.

### 2.7. Biofilm Biomass Quantification

The cellular density was adjusted to 1 × 10^7^ yeast/mL in RPMI for the 29 isolates of* C. albicans* and the reference strain, and the cells were then added to a 96-well plate [22]. For biofilm formation, the microtiter plates were incubated for 24 h at 35°C with shaking (60 rpm). The microtiter plates were then washed once in PBS (0.1 M, pH 7) to remove loosely attached cells. Biofilm formation was then assessed by quantifying the total biomass using crystal violet staining [23]. The optical density (OD) was then determined with a spectrophotometer (Q798DRM, Quimis, Diadema, Brazil) at 570 nm. The experiments were performed in triplicate.

### 2.8. Assessment of Antibiofilm Activity of the Propolis Extractive Solution

The effect of PES on biofilm formation was evaluated similarly to the biofilm assay, with minor modifications. The PES at 0.5 × MIC (273.43 *μ*g/mL) was added simultaneously to the addition of the 29 isolates and reference strain in a 96-well plate. To form biofilms, the microtiter plates were incubated for 24 h at 35°C with shaking (60 rpm). The microtiter plates were washed with PBS to remove loosely attached cells. Afterward, biofilm formation was assessed by quantifying the total biomass using crystal violet staining [23], and the OD was read on a spectrophotometer at 570 nm. The experiments were performed in triplicate.

#### 2.8.1. *Candida albicans* Biofilm Characterization

Biofilm characterization was performed using (i)* Candida albicans* viability assays to determine colony-forming units (CFUs), (ii) protein and carbohydrate quantification of the biofilm matrix, and (iii) scanning electron microscopy (SEM). The* C. albicans* clinical isolates were A2 and 31MC, which were chosen randomly, and the reference strain. The concentration of PES was 273.43 *μ*g/mL (0.5 × MIC).

#### 2.8.2. *Candida albicans* Viability Assays

The number of cultivable cells is expressed as CFU/mL. Briefly, the same procedure as the one for biofilm formation was performed with and without exposure to PES but before staining with crystal violet. Phosphate-buffered saline (200 *μ*L) was added to each well. The wells were then scraped. The complete removal of adhered cells was confirmed by crystal violet staining. The obtained suspensions were vortexed vigorously for 5 min, and then serial dilutions in PBS were subcultured onto SDA and incubated for 24 h at 35°C to determine CFU/mL. The determination of CFUs was performed in triplicate [24].

#### 2.8.3. Quantification of Proteins and Carbohydrates in Biofilm Matrix

For the analysis of matrix material, biofilms were formed in 24-well polystyrene microtiter plates (Techno Plastic Products, Switzerland). For this, 1 mL of the yeast cell suspension (1 × 10^7^ cells/mL in RPMI) with or without PES was added to each well, and biofilms were formed as described previously. After 24 h, the biofilm matrix was extracted using a slight modification of a previously described protocol [24]. Briefly, the biofilm samples were scraped from the 24-well plates, resuspended with ultra-pure water, and sonicated (Sonic Dismembrator Ultrasonic Processor, Fisher Scientific) for 45 s at 30 W, and then the suspension was vortexed for 2 min. The suspension was centrifuged at 3000 ×g for 10 min at 4°C, and the supernatant was filtered through a 0.2 mm nitrocellulose filter and stored at −20°C until analysis. Proteins and carbohydrates were measured using a Nano Drop spectrophotometer (Nano Drop 2000 UV-Vis Spectrophotometer, Thermo Scientific, Wilmington, DE, USA). The experiments were performed in triplicate and in three independent assays.

#### 2.8.4. Scanning Electron Microscopy

Biofilms for SEM were formed in 24-well polystyrene microtiter plates (Techno Plastic Products, Switzerland), in which 1 mL of the yeast cell suspension (1 × 10^7^ cells/mL in RPMI) with and without PES was added to each well. The biofilms were then formed as described previously. The plate wells were washed with sterile PBS. The plate was allowed to air-dry. Glutaraldehyde (2.5%) was then added for fixation for 2 h. After fixation, the cells were dehydrated with a series of ethanol washes (70, 80, 90, 95, and 100%). The surface of the well was cut and fixed on supports, critical-point dried in CO_2_, coated with gold-palladium under argon atmosphere using a gold sputter module in a high-vacuum evaporator. Samples were then observed with Shimazu SS-550 Super scan (SHIMADZU, Tokyo, Japan) at magnifications of 350x, 1000x, and 4000x [24].

### 2.9. Statistical Analysis

The data were analyzed using Prism 6.0 software (GraphPad, San Diego, CA, USA). One-way analysis of variance (ANOVA) with the Bonferroni test was used. All of the tests were performed with a confidence level of 95%. Values of *P* ≤ 0.05 were considered statistically significant.

## 3. Results

### 3.1. Chemical Composition of the Propolis Extractive Solution

Propolis was collected in North Region of Paraná State (Brazil). Even presenting biochemically complicated substances, propolis of this place is well studied and chemically characterized, as well as its ethanolic extractive solutions [13, 14, 18].

Phenolic compounds may be simple or complex structures [25], and they may be isolated from ethanolic extracts from different natural sources, such as plants, lichens, and macroscopic fungi [26]. It is a class of compounds, originates from the secondary metabolism of plants, and has anti-inflammatory, antimicrobial, and particularly antifungal activities [27–29]. The flavonoids constitute a very important class of polyphenols, widely present in propolis, to which the greatest part of propolis biological activities is attributed [8]. The quality control of PES was performed according to techniques approved by Farmacopéia [30] and described in scientific studies [14]. The results obtained with regard to dryness residue, relative density, pH, and TPC are displayed in Table 1. These results showed that the quality of PES [16] was adequate for the present study.

### 3.2. Propolis Extractive Solution Activity against* C. albicans* Isolated from VVC

The results of MICs for the 29* C. albicans* isolated from VVC and the reference strain are presented in Figure 2. All of the* C. albicans* strains were inhibited by PES, with MICs that ranged from 68.35 to 546.87 *μ*g/mL. The MIC_50_ (i.e., the MIC that was able to inhibit 50% of the isolates tested) and MIC_90_ (i.e., the MIC that was able to inhibit 90% of the isolates tested) corresponded to 546.87 *μ*g/mL. Based on these results, the MFC was also determined by fungicidal activity (Figure 3). The MFC test presented the same value as the one found for the MIC (546.87 *μ*g/mL).

The MICs of fluconazole and nystatin for the* C. albicans* isolates and reference strain are presented in Table 2. All 29 isolates were susceptible to fluconazole, with MICs that ranged from 0.125 to 8 *μ*g/mL; (MIC_50_) and (MIC_90_) were 0.125 and 1.0 *μ*g/mL, respectively. For nystatin, 75% of the clinical isolates were susceptible, and 25% were DDS. The MICs ranged from 0.125 to 8 *μ*g/mL, and the MIC_50_ and MIC_90_ were 0.125 and 8 *μ*g/mL, respectively.

### 3.3. Effect of the Propolis Extractive Solution on Human Cervical Cell Monolayer Viability

**Figure 1:**
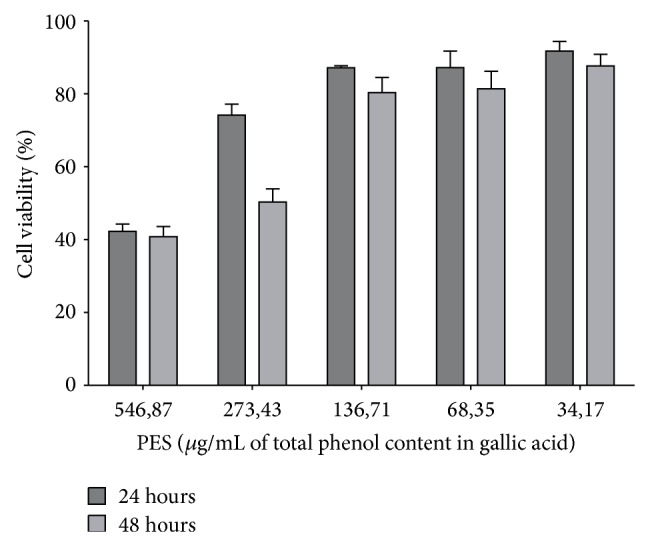
Cell viability 24 and 48 h after exposure to concentrations of PES.

**Figure 2:**
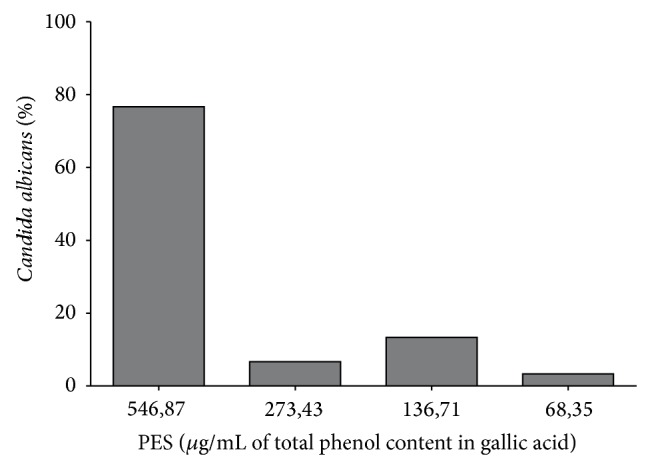
Susceptibility of* Candida albicans* to PES (*μ*g/mL of total phenol content in gallic acid) from 29 vaginal isolates and the reference strain.

**Figure 3:**
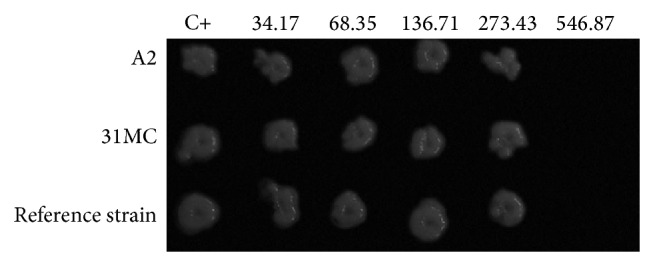
Example of plate bioassay to determine minimum fungicidal concentration (MFC) in* Candida albicans* (clinical isolates) and ATCC90028 (reference strain). Foot note: C+: positive control (*Candida albicans* without PES); reference strain: ATCC90028; concentrations are in *μ*g/mL of total phenol content (expressed in gallic acid).

Human cervical cells showed 42.24% cell viability after 24 h exposure to PES at concentration tested in the susceptibility tests (546.87 *μ*g/mL), whereas cell viability was 91.72% at the lower concentration (34.17 *μ*g/mL) according to Figure 1. After 48 h exposure to PES, cell viabilities were 40.82% and 87.65% for 546.87 *μ*g/mL and 34.17 *μ*g/mL, respectively. For most of the concentrations (60%), no statistically significant difference in cell viability was found at 24 or 48 h (*P* ≤ 0.05).

### 3.4. Biofilm Biomass Formation on Abiotic Surface

All 29 clinical isolates and the reference strain were able to form a biofilm biomass with 24 h incubation. However, variability in the biofilms was observed among the clinical isolates, which ranged from 0.53 to 12.11 Abs/cm^2^. The average OD of the biofilm was 3.73 Abs/cm^2^ (Table 3).

### 3.5. Effect of the Propolis Extractive Solution on Biofilm

As shown in Table 3, PES was able to decrease biofilm biomass formation in most of the isolates (93.34%) compared with the control group that was not exposed to PES. This reduction ranged from 26.44% to 95.35%. Only 6.66% (2/30) of the isolates exhibited an increase in biofilm formation after exposure to PES.

**Figure 4:**
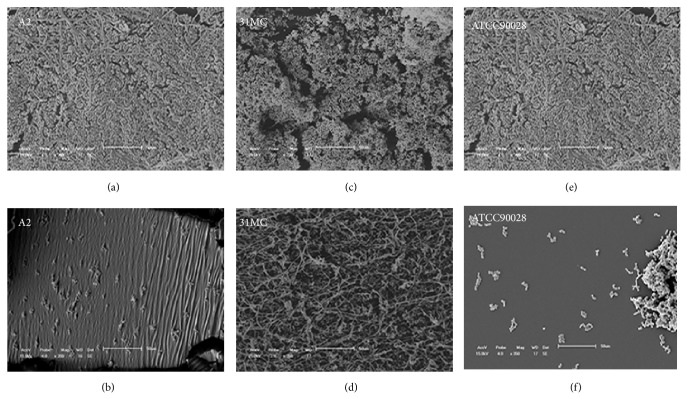
Scanning electron microscopy images of the effect of the PES on* Candida albicans* biofilm on a polystyrene surface for three samples: A2 isolate ((a), (b)), 31MC isolate ((c), (d)), and ATCC90028 reference strain ((e), (f)). Biofilms without the PES ((a), (c), and (e)) and biofilms with the PES (273.43 *μ*g/mL of total phenol content in gallic acid) after 24 h ((b), (d), and (f)) at 350x magnification.

To better understand the action of PES on biofilm formation and matrix composition, two clinical isolates (A2 and 31MC) and the reference strain were randomly chosen to analyze the characteristics of the biofilm (Table 4). SEM was used to examine the biofilm structure before and after exposure to PES; observing the morphological characteristics of* C. albicans* was possible (Figure 4). After exposure to PES, the mature biofilms showed a dense network of cells with various morphologies. The biofilms of the A2 isolate and reference strains were composed of both yeast and pseudohyphae and formed multilayer, compact biofilms that covered the entire surface. In contrast, the biofilm of the 31MC isolate was devoid of pseudohyphae and consisted of noncontiguous cell aggregates. After exposure to PES, we observed a decrease in the OD for the A2 isolate and reference strain (Table 3). SEM showed a marked reduction of this biofilm. Interestingly, the 31MC isolate exhibited an increase in OD, but SEM did not indicate an increase in cells after exposure to PES.

A reduction of the biofilm CFUs was observed for all of the isolates after exposure to PES compared with the control (i.e., biofilm formation without PES), which was statistically significant (*P* ≤ 0.05) for the A2 isolate (61.69%) and reference strain (56.70%). Additionally, the A2 isolate exhibited 44.33% reduction of carbohydrates, and total protein was maintained. For the reference strain, a significant reduction was found for all of the analyses (*P* ≤ 0.05). The CFUs were reduced by more than 50%, and carbohydrates and proteins were reduced by approximately 70% and 90%, respectively (Table 4).

For the 31MC isolate of* C. albicans*, the presence of PES also reduced the CFUs in the biofilm by 4.20%, but this reduction was not statistically significant (*P* ≤ 0.05). We observed a reduction of the biofilm matrix, reflected by a 50% reduction of carbohydrates and 80% reduction of proteins.

## 4. Discussion

The incidence of VVC has increased in recent years, and* C. albicans* is still the most prevalent species [1]. The different forms of VVC have a significant effect on quality of life and a substantial impact on healthcare systems. The clinical treatment of VVC is routinely performed with polyenes or azole derivatives. However, these drugs have undesirable side effects and toxicity. Moreover, the resistance of* C. albicans* to polyenes and azole derivatives has been described [18]. Exacerbation of the virulence of* C. albicans* by biofilm formation enhances the infectivity of VVC, which confers resistance to antifungal therapy and the ability of the cells that are inside the biofilm to resist immune system defenses [31, 32]. The limited number of antifungal drugs that are available for treatment combined and the continuous increase in the incidence of* C. albicans* infection have necessitated the search for novel treatment and prevention strategies. Thus, the present study evaluated the* in vitro* effect of PES as a possible antifungal drug and antibiofilm agent.

Natural products with antifungal activity have been discovered [33–36]. Propolis has received the attention of clinicians and researchers because of its diverse pharmacological activities and low toxicity [37–39].

Our first step was to evaluate the susceptibility of clinical isolates from VVC to antifungals that are routinely used in clinical practice. As shown in Table 2,* C. albicans* isolates from VVC were susceptible to fluconazole, but 25% of the isolates showed resistance to nystatin. Similar results were reported by Dalben-Dota et al. [18]. The PES inhibited the growth of all of the strains tested, with a MIC of 546.87 *μ*g/mL. Importantly, the complete inhibition of growth and death occurred even for clinical isolates with DDS to nystatin, suggesting a better antifungal action than the independent drugs tested against the isolates tested.

In addition to being effective against microorganisms, a drug must also show low cytotoxicity for clinical applicability. Propolis varies according to the geographic region where it is extracted [7]. Based on the MIC results for PES, we evaluated cytotoxicity in HeLa cells at the MIC, 0.5 × MIC, 0.25 × MIC, 0.125 × MIC, and 0.06 × MIC. The viability of HeLa cells was satisfactory for more than 80% of these concentrations at 24 and 48 h (Figure 1). Therefore, PES used in the present study demonstrated low toxicity in human cells, which has also been reported by other authors who worked with PESs of different origins [40]. Research indicates that PES can be a good treatment alternative for chronic vaginitis [9]. Moreover,* in vitro* and* in vivo* studies have focused on using PES in pharmaceutical formulations that retain its properties, including mucoadhesive gels [41] and mucoadhesive systems that contain thermoresponsive PES [42], for the possible treatment of VVC.

Biofilm formation in* Candida* species, in addition to possibly being a key factor in the survival of this species, may also be responsible for their being particularly well adapted to the colonization of tissues and indwelling devices. In VVC, biofilm may be closely related to RVVC and therefore the resistance to antifungal therapy. This could be attributed to biofilm formation on medical devices, like IUD [11]. Therefore, biofilm formation on surfaces is a key attribute of the pathogenicity of* Candida* spp. and a major challenge for the treatment of* Candida* infections in related biomaterials [43]. The possible mechanisms of biofilm resistance to antimicrobial agents include limited drug penetration through the extracellular matrix, phenotypic changes, induction of the expression of resistance genes, and a small number of “resistant” cells [44].

Therefore, in the present study, our next step was to evaluate the biofilm formation ability of* C. albicans* from VVC (Table 3). All of the isolates studied herein formed biofilms on polystyrene surfaces under the assayed conditions, and this ability was highly strain-dependent. These results reflect inherent differences in the clinical isolates and may be related to potential pathogenicity. Furthermore, intra- and interspecific variability with regard to the ability of* Candida* species to form biofilms has been observed [45]. In fact, SEM revealed structural and morphological differences in the biofilms between the studied strains.

Based on the biofilm formation that was observed and the effect of PES on* C. albicans* from VVC, we evaluated the influence of PES at 0.5 × MIC during biofilm formation. Generally, PES inhibited biofilm formation in 93.34% (28/30) of the strains tested (*P* ≤ 0.05) and inhibited the biofilm formation of DDS isolates to nystatin. This reduction of biofilm formation by PES has been previously reported, but the previous study analyzed others parameters, such as metabolic activity,* in vitro* [46].

One of the most important characteristics of fungal biofilms is the presence and composition of the extracellular matrix [47]. Therefore, to better understand the influence of PES on* C. albicans* biofilm, we performed SEM and assessed the cell viability, protein, and carbohydrate characteristics of the biofilm (Table 4). The biofilms of A2 isolate and reference strain were composed of yeast and pseudohyphae and formed multilayer, compact biofilms that covered the entire surface. After exposure to PES, SEM revealed a marked reduction of these biofilms. The results demonstrated that both strains (A2 and reference) exhibited a significant decrease in CFUs (*P* ≤ 0.05). Furthermore, the biofilm of the reference strain exhibited reductions of the biofilm biomass, carbohydrates, and proteins (*P* ≤ 0.05). Another study demonstrated the efficient action of the ethanolic extract of three types of propolis on planktonic and biofilm cells of* Candida* species and observed the antibiofilm action of PES, reflected by a reduction of the biofilm formed by yeast [48].

Interestingly, the biofilm of the 31MC isolate presented a multilayer, compact biofilm that covered the entire surface. After exposure to PES, the biofilm matrix exhibited a significant reduction of carbohydrates and proteins. The increase in OD revealed by crystal violet staining was justified by the observation of filamentation, which was visible only under a SEM. The increase in biofilm was justified by yeast filamentation and possibly occurred as a response of* C. albicans* to environmental stress [10, 49], which was, in this case, exposure to PES.

Notwithstanding the observations that PES reduced the matrix and/or number of cells of* C. albicans* in the biofilm, PES may have affected the structure of* C. albicans*. According to the literature, the deformation of the biofilm implies greater permeability of the drug and consequently a reduction of the resistance and infectivity of the clinical isolates [19].

## 5. Conclusions

Our results support the already described limited effectiveness of nystatin. Despite the susceptibility of the clinical isolates to fluconazole, the present results demonstrated the increasing resistance of* C. albicans* to this azole. The PES had antifungal activity and may be a useful antibiofilm product that addresses the problem of drug resistance and RVVC associated with the biofilm growth of* C. albicans*. Further research should be extended to biotic surfaces. The present study contributes to a better understanding of the antibiofilm action of propolis and helps elucidate the development of RVVC related to the use of IUDs and biofilm formation.

## Figures and Tables

**Table 1:** Physicochemical characteristics of propolis extractive solution (PES).

Parameters	Average	SD	RSD (%)
Relative density (g/mL)	0.8718	0.0008	0.09
pH value	5.31	0.0115	0.22
Dryness residue (%, w/w)	17.11	0.5733	3.35
Total phenol content (%, w/v)	4.07	0.0806	1.98

SD, standard deviation; RSD (%), relative standard deviation.

**Table 2:** Minimum inhibitory concentration (MIC) values (*µ*g/mL) for fluconazole and nystatin against 29 vaginal isolates of *Candida albicans *and the reference strain.

*C. albicans*	MIC (*µ*g/mL)^a,b^											MIC_50_	MIC_90_
	*n*	0,125	0,25	0,5	1,0	2,0	4,0	8,0	16,0	32,0	64,0		
Fluconazole	30	18	7	1	3	—	—	1	—	—	—	0,125	1,0
Nystatin	30	18	1	—	—	2	2	7	—	—	—	0,125	8,0

^a^MIC of the fluconazole: the lowest concentration of the drug that could inhibit 50% of the growth of each yeast.

^b^MIC of the nystatin: the lowest concentration of the drug that could inhibit 90% of the growth of each yeast.

MIC_50_ and MIC_90_: MIC of fluconazole/nystatin that could inhibit 50% and 90% of the growth of the isolates, respectively.

**Table 3:** Effect of PES on biofilm biomass for the 29 samples of *Candida albicans *and the reference strain isolated from VVC.

Samples	Biofilm without PES (Abs/cm^2^)	Biofilm with PES (Abs/cm^2^)	Reduction (%)
A2^*^	2,37	0,11	95,35
B11^*^	12,11	0,76	93,72
D4	1,91	0,54	71,72
F9	2,15	0,24	88,83
F10^*^	5,36	0,73	86,38
F12	4,07	0,97	76,16
G23^*^	2,68	0,23	91,41
H1^*^	4,32	0,32	92,59
H5	2,21	0,79	64,25
I1^*^	8,76	4,07	53,53
I10^*^	2,48	0,65	73,79
I14	1,08	0,40	62,69
61KD^*^	3,28	0,62	81,09
109KD	1,00	0,27	73,00
110KD^*^	5,19	2,07	60,11
111KD	1,21	0,89	26,44
112KD	2,02	1,38	31,68
117KD	2,91	2,03	30,24
119KD	2,56	1,40	45,31
126KD^*^	6,74	2,39	64,54
132KD^*^	5,84	1,09	81,33
134KD^*^	2,79	0,20	92,83
73D	2,77	4,14	0
1MG^*^	5,35	1,97	63,17
6MG^*^	4,72	1,11	76,48
21MG	2,46	1,55	37,00
3MC^*^	3,06	0,99	67,64
31MC^*^	2,90	3,28	0
100MC^*^	6,98	1,02	85,38
ATCC90028^*^	0,53	0,35	33,96

Means	3,73	1,22	63,35

The values are means. ^*^Significantly different (*P* < 0.05) among biofilm without PES and with PES.

**Table 4:** Effect of PES on established *Candida albicans* biofilms.

Samples	CFU/cm^2^		Proteins (ng/mL)		Carbohydrates (ng/mL)	
	C	PES	C	PES	C	PES
A2	3,08 × 10^4^	1,18 × 10^4^	0,03	0,04	0,75	0,44
31MC	4,05 × 10^4^	3,88 × 10^4^	0,05	0,01	1,10	0,58
ATCC90028	6,42 × 10^4^	2,78 × 10^4^	0,03	0,01	0,49	0,06

C: control = without PES.

PES: 273,43 *µ*g/mL of total phenol content in gallic acid.
